# 1-[2-(Pyrazin-2-ylsulfan­yl)eth­yl]pyrazine-2(1*H*)-thione

**DOI:** 10.1107/S1600536811001474

**Published:** 2011-01-15

**Authors:** Xi-Zai Zhao, Jing-Jing Jia, Miao Ou-Yang, Fu-Ping Huang, Yi-Min Jiang

**Affiliations:** a College of Chemistry and Chemical Engineering, Guangxi Normal University, Guilin, Guangxi 541004, People’s Republic of China; b Department of Chemistry and Life Science, Hechi University, Yizhou, Guangxi 546300, People’s Republic of China

## Abstract

The title multifunctional twisted organic ligand, C_10_H_10_N_4_S_2_, contains a short C=S bond [1.671 (2) Å]. The dihedral angle between the two pyrazine rings is 39.83 (6)°. In the crystal, inter­molecular C—H⋯N and C—H⋯S hydrogen bonds result in the formation of a supra­molecular network.

## Related literature

The assembly of mol­ecular units in predefined arrangements is a key goal in the synthesis of crystal structures by design, see: Zheng *et al.* (2003 ▶). For bond lengths and angles in the ligand, see: Etter *et al.* (1992 ▶). For a description of the Cambridge Structural Database, see: Allen (2002 ▶). For versatile ligands, see: Devel *et al.* (2003 ▶).
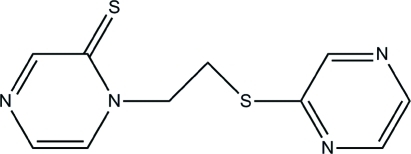


## Experimental

### 

#### Crystal data

C_10_H_10_N_4_S_2_
*M* *_r_* = 250.34
Monoclinic, 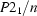

*a* = 9.762 (7) Å
*b* = 11.737 (9) Å
*c* = 10.129 (8) Å
β = 92.628 (9)°
*V* = 1159.2 (15) Å^3^
*Z* = 4
Mo *K*α radiation
μ = 0.44 mm^−1^
*T* = 296 K
0.32 × 0.20 × 0.08 mm

#### Data collection

Bruker SMART CCD area-detector diffractometer
Absorption correction: multi-scan (*SADABS*; Bruker, 1997 ▶) *T*_min_ = 0.873, *T*_max_ = 0.966
6280 measured reflections
2160 independent reflections
1879 reflections with *I* > 2σ(*I*)
*R*_int_ = 0.024

#### Refinement

*R*[*F* ^2^ > 2σ(*F* ^2^)] = 0.032
*wR*(*F* ^2^) = 0.085
*S* = 1.06
2160 reflections
145 parameters
H-atom parameters constrained
Δρ_max_ = 0.22 e Å^−3^
Δρ_min_ = −0.22 e Å^−3^

### 

Data collection: *SMART* (Bruker, 1997 ▶); cell refinement: *SAINT* (Bruker, 1997 ▶); data reduction: *SAINT*; program(s) used to solve structure: *SHELXS97* (Sheldrick, 2008 ▶); program(s) used to refine structure: *SHELXL97* (Sheldrick, 2008 ▶); molecular graphics: *SHELXTL* (Sheldrick, 2008 ▶); software used to prepare material for publication: *SHELXTL*.

## Supplementary Material

Crystal structure: contains datablocks I, global. DOI: 10.1107/S1600536811001474/bv2167sup1.cif

Structure factors: contains datablocks I. DOI: 10.1107/S1600536811001474/bv2167Isup2.hkl

Additional supplementary materials:  crystallographic information; 3D view; checkCIF report

## Figures and Tables

**Table 1:** Hydrogen-bond geometry (Å, °)

*D*—H⋯*A*	*D*—H	H⋯*A*	*D*⋯*A*	*D*—H⋯*A*
C4—H4⋯N4^i^	0.93	2.56	3.298 (3)	137
C2—H2⋯S2^ii^	0.93	2.99	3.544 (3)	119
C3—H3⋯S2^iii^	0.93	2.92	3.741 (3)	148
C8—H8⋯S1^iv^	0.93	2.97	3.738 (3)	140
C9—H9⋯S1^v^	0.93	2.98	3.897 (3)	169

Symmetry codes: (i) 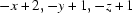; (ii) 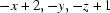; (iii) 
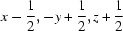
; (iv) 
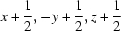
; (v) 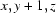.

**Fig. 1.**
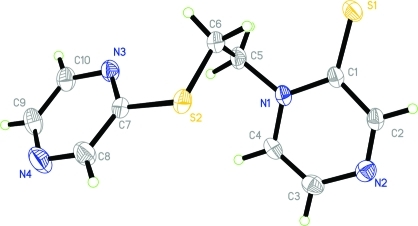
The molecular structure of the title molecule, showing the atom-numbering scheme.

**Fig. 2.**
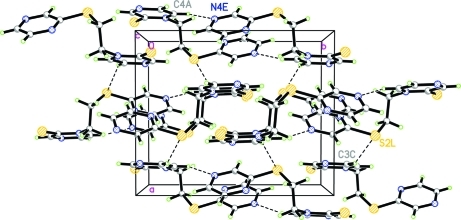
A packing diagram for (I). Hydrogen bonds are shown as dashed lines.

## supplementary crystallographic information

### Comment

In the synthesis of crystal structures by design, the assembly of molecular units in predefined arrangements is a key goal (Zheng *et al.*, 2003). Pyrazine-2-thiol is a versatile ligand which may adopt a variety of bonding modes in its coordination compounds. We originally attempted to synthesize complexes featuring Co metal chains by reaction of the Co(II) ion with pyrazine-2-thiol ligand. Unfortunately, we obtained only the title ligand, and we report herein its crystal structure. In  I (Fig. 1), the ligand bond lengths and angles are within normal ranges (Etter *et al.*, 1992). The C=S bond length was compared with the mean C—S and C=S bond lengths retrieved from a search of thiol and thione crystal structures from the CSD (Allen, 2002). X-ray data from I give a C=S bond length of 1.671 (2) Å, which is shorter than the value of 1.698 (2) Å for 2-thiopyridone and indicates a C=S rather than C-S bond. In the crystal structure, intermolecular C—H···N and C—H···S hydrogen bonds (Table 1 and Fig. 2) result in the formation of a supramolecular network structure.

### Experimental

For the preparation of (I), a solution of CoCl_2_.6H_2_O (47.9 mg, 0.2 mmol) in 5 ml distilled water was added dropwise to a solution of pyrazine-2-thiol (250 mg, 2 mmol) and 1,2-dibromoethane (0.09 ml, 1 mmol) in 15 ml sodium ethoxide/ethanol solution. The resulting solution was stirred at 352 K for 3 h, then cooled to room temperature and filtered. A yellow-block crystal suitable for X-ray diffraction were obtained by slow evaporation after several days.

### Refinement

H atoms were positioned geometrically, with C—H = 0.93 and 0.97 Å for aromatic and methyl H atoms, and constrained to ride on their parent atoms, with *U*_iso_(H) = 1.2*U*_eq_(C)

### Crystal data

**Table:**

C_10_H_10_N_4_S_2_	*F*(000) = 520
*M**_r_* = 250.34	*D*_x_ = 1.434 Mg m^−^^3^
Monoclinic, *P*2_1_/*n*	Mo *K*α radiation, λ = 0.71073 Å
*a* = 9.762 (7) Å	Cell parameters from 3231 reflections
*b* = 11.737 (9) Å	θ = 2.7–27.9°
*c* = 10.129 (8) Å	µ = 0.44 mm^−^^1^
β = 92.628 (9)°	*T* = 296 K
*V* = 1159.2 (15) Å^3^	Block, yellow
*Z* = 4	0.32 × 0.20 × 0.08 mm

### Data collection

**Table:**

Bruker SMART CCD area-detector diffractometer	2160 independent reflections
Radiation source: fine-focus sealed tube	1879 reflections with *I* > 2σ(*I*)
graphite	*R*_int_ = 0.024
φ and ω scans	θ_max_ = 25.5°, θ_min_ = 2.7°
Absorption correction: multi-scan (*SADABS*; Bruker, 1997)	*h* = −11→11
*T*_min_ = 0.873, *T*_max_ = 0.966	*k* = −14→12
6280 measured reflections	*l* = −12→10

### Refinement

**Table:**

Refinement on *F*^2^	Primary atom site location: structure-invariant direct methods
Least-squares matrix: full	Secondary atom site location: difference Fourier map
*R*[*F*^2^ > 2σ(*F*^2^)] = 0.032	Hydrogen site location: inferred from neighbouring sites
*wR*(*F*^2^) = 0.085	H-atom parameters constrained
*S* = 1.06	*w* = 1/[σ^2^(*F*_o_^2^) + (0.039*P*)^2^ + 0.347*P*] where *P* = (*F*_o_^2^ + 2*F*_c_^2^)/3
2160 reflections	(Δ/σ)_max_ = 0.001
145 parameters	Δρ_max_ = 0.22 e Å^−^^3^
0 restraints	Δρ_min_ = −0.22 e Å^−^^3^

### Special details

**Table:**

Geometry. All e.s.d.'s (except the e.s.d. in the dihedral angle between two l.s. planes) are estimated using the full covariance matrix. The cell e.s.d.'s are taken into account individually in the estimation of e.s.d.'s in distances, angles and torsion angles; correlations between e.s.d.'s in cell parameters are only used when they are defined by crystal symmetry. An approximate (isotropic) treatment of cell e.s.d.'s is used for estimating e.s.d.'s involving l.s. planes.
Refinement. Refinement of *F*^2^ against ALL reflections. The weighted *R*-factor *wR* and goodness of fit *S* are based on *F*^2^, conventional *R*-factors *R* are based on *F*, with *F* set to zero for negative *F*^2^. The threshold expression of *F*^2^ > σ(*F*^2^) is used only for calculating *R*-factors(gt) *etc*. and is not relevant to the choice of reflections for refinement. *R*-factors based on *F*^2^ are statistically about twice as large as those based on *F*, and *R*- factors based on ALL data will be even larger.

### Fractional atomic coordinates and isotropic or equivalent isotropic displacement parameters (Å^2^)

**Table:**

	*x*	*y*	*z*	*U*_iso_*/*U*_eq_	
C1	0.86032 (16)	0.00044 (13)	0.30816 (17)	0.0373 (4)	
C2	0.83823 (17)	−0.05822 (14)	0.42873 (18)	0.0418 (4)	
H2	0.8326	−0.1372	0.4242	0.050*	
C3	0.83014 (18)	0.10299 (16)	0.54879 (19)	0.0464 (4)	
H3	0.8205	0.1393	0.6295	0.056*	
C4	0.84852 (16)	0.16689 (14)	0.44097 (18)	0.0413 (4)	
H4	0.8502	0.2459	0.4481	0.050*	
C5	0.8960 (2)	0.18947 (15)	0.20807 (19)	0.0460 (4)	
H5A	0.8572	0.2646	0.2203	0.055*	
H5B	0.8538	0.1572	0.1280	0.055*	
C6	1.0492 (2)	0.20010 (15)	0.19297 (18)	0.0479 (4)	
H6A	1.0649	0.2399	0.1111	0.058*	
H6B	1.0879	0.1243	0.1861	0.058*	
C7	1.08904 (17)	0.41615 (14)	0.29421 (17)	0.0373 (4)	
C8	1.1302 (2)	0.49827 (17)	0.3863 (2)	0.0593 (5)	
H8	1.1800	0.4757	0.4623	0.071*	
C9	1.0294 (2)	0.63363 (17)	0.2595 (2)	0.0587 (5)	
H9	1.0053	0.7093	0.2443	0.070*	
C10	0.99020 (19)	0.55385 (15)	0.1681 (2)	0.0494 (5)	
H10	0.9419	0.5771	0.0915	0.059*	
N1	0.86480 (13)	0.11696 (11)	0.32118 (14)	0.0366 (3)	
N2	0.82515 (15)	−0.01237 (13)	0.54431 (15)	0.0464 (4)	
N3	1.01858 (15)	0.44304 (12)	0.18458 (15)	0.0435 (4)	
N4	1.1012 (2)	0.60692 (15)	0.3698 (2)	0.0706 (5)	
S2	1.13825 (5)	0.27417 (4)	0.32673 (5)	0.04440 (15)	
S1	0.87993 (6)	−0.06784 (4)	0.16546 (5)	0.05616 (17)	

### Atomic displacement parameters (Å^2^)

**Table:**

	*U*^11^	*U*^22^	*U*^33^	*U*^12^	*U*^13^	*U*^23^
C1	0.0380 (9)	0.0282 (8)	0.0447 (9)	−0.0010 (6)	−0.0081 (7)	−0.0002 (7)
C2	0.0419 (9)	0.0322 (9)	0.0504 (11)	−0.0052 (7)	−0.0058 (8)	0.0033 (7)
C3	0.0401 (9)	0.0512 (11)	0.0483 (10)	−0.0018 (8)	0.0067 (8)	−0.0111 (9)
C4	0.0360 (9)	0.0328 (9)	0.0549 (11)	0.0003 (7)	0.0008 (8)	−0.0097 (8)
C5	0.0614 (11)	0.0279 (9)	0.0477 (10)	−0.0020 (8)	−0.0083 (9)	0.0071 (7)
C6	0.0671 (12)	0.0311 (9)	0.0463 (10)	−0.0027 (8)	0.0097 (9)	0.0012 (7)
C7	0.0380 (9)	0.0327 (8)	0.0415 (9)	−0.0037 (7)	0.0035 (7)	0.0037 (7)
C8	0.0782 (14)	0.0452 (11)	0.0527 (12)	−0.0052 (10)	−0.0172 (10)	−0.0010 (9)
C9	0.0630 (12)	0.0311 (10)	0.0815 (15)	0.0021 (8)	−0.0012 (11)	0.0003 (10)
C10	0.0481 (10)	0.0384 (10)	0.0610 (12)	0.0015 (8)	−0.0052 (9)	0.0091 (8)
N1	0.0382 (7)	0.0266 (7)	0.0445 (8)	0.0006 (5)	−0.0043 (6)	0.0001 (6)
N2	0.0426 (8)	0.0493 (9)	0.0473 (9)	−0.0054 (7)	0.0015 (7)	0.0029 (7)
N3	0.0496 (9)	0.0339 (8)	0.0464 (9)	−0.0016 (6)	−0.0042 (7)	0.0035 (6)
N4	0.0953 (14)	0.0412 (10)	0.0735 (13)	−0.0043 (9)	−0.0152 (11)	−0.0117 (9)
S2	0.0490 (3)	0.0346 (3)	0.0494 (3)	−0.00021 (18)	0.0004 (2)	0.01038 (18)
S1	0.0878 (4)	0.0359 (3)	0.0439 (3)	0.0014 (2)	−0.0074 (3)	−0.00664 (19)

### Geometric parameters (Å, °)

**Table:**

C1—N1	1.374 (2)	C6—S2	1.800 (2)
C1—C2	1.427 (3)	C6—H6A	0.9700
C1—S1	1.671 (2)	C6—H6B	0.9700
C2—N2	1.300 (2)	C7—N3	1.317 (2)
C2—H2	0.9300	C7—C8	1.388 (3)
C3—C4	1.344 (3)	C7—S2	1.761 (2)
C3—N2	1.356 (3)	C8—N4	1.315 (3)
C3—H3	0.9300	C8—H8	0.9300
C4—N1	1.363 (2)	C9—N4	1.328 (3)
C4—H4	0.9300	C9—C10	1.359 (3)
C5—N1	1.470 (2)	C9—H9	0.9300
C5—C6	1.515 (3)	C10—N3	1.339 (2)
C5—H5A	0.9700	C10—H10	0.9300
C5—H5B	0.9700		
			
N1—C1—C2	113.79 (15)	S2—C6—H6B	108.7
N1—C1—S1	123.76 (13)	H6A—C6—H6B	107.6
C2—C1—S1	122.45 (14)	N3—C7—C8	121.60 (17)
N2—C2—C1	126.58 (17)	N3—C7—S2	120.77 (13)
N2—C2—H2	116.7	C8—C7—S2	117.60 (15)
C1—C2—H2	116.7	N4—C8—C7	122.3 (2)
C4—C3—N2	122.43 (17)	N4—C8—H8	118.9
C4—C3—H3	118.8	C7—C8—H8	118.9
N2—C3—H3	118.8	N4—C9—C10	122.09 (19)
C3—C4—N1	120.59 (16)	N4—C9—H9	119.0
C3—C4—H4	119.7	C10—C9—H9	119.0
N1—C4—H4	119.7	N3—C10—C9	122.29 (19)
N1—C5—C6	111.51 (15)	N3—C10—H10	118.9
N1—C5—H5A	109.3	C9—C10—H10	118.9
C6—C5—H5A	109.3	C4—N1—C1	120.57 (14)
N1—C5—H5B	109.3	C4—N1—C5	118.78 (15)
C6—C5—H5B	109.3	C1—N1—C5	120.53 (14)
H5A—C5—H5B	108.0	C2—N2—C3	116.02 (16)
C5—C6—S2	114.12 (13)	C7—N3—C10	115.77 (16)
C5—C6—H6A	108.7	C8—N4—C9	115.98 (18)
S2—C6—H6A	108.7	C7—S2—C6	101.42 (9)
C5—C6—H6B	108.7		
			
N1—C1—C2—N2	−1.3 (3)	C6—C5—N1—C4	92.37 (18)
S1—C1—C2—N2	178.29 (14)	C6—C5—N1—C1	−83.77 (19)
N2—C3—C4—N1	−0.8 (3)	C1—C2—N2—C3	1.6 (3)
N1—C5—C6—S2	−65.89 (18)	C4—C3—N2—C2	−0.5 (3)
N3—C7—C8—N4	0.2 (3)	C8—C7—N3—C10	0.2 (3)
S2—C7—C8—N4	178.53 (18)	S2—C7—N3—C10	−178.07 (13)
N4—C9—C10—N3	1.5 (3)	C9—C10—N3—C7	−1.0 (3)
C3—C4—N1—C1	1.1 (2)	C7—C8—N4—C9	0.2 (3)
C3—C4—N1—C5	−175.01 (16)	C10—C9—N4—C8	−1.0 (3)
C2—C1—N1—C4	−0.2 (2)	N3—C7—S2—C6	−7.14 (16)
S1—C1—N1—C4	−179.72 (12)	C8—C7—S2—C6	174.49 (16)
C2—C1—N1—C5	175.90 (15)	C5—C6—S2—C7	−74.03 (14)
S1—C1—N1—C5	−3.6 (2)		

### Hydrogen-bond geometry (Å, °)

**Table:**

*D*—H···*A*	*D*—H	H···*A*	*D*···*A*	*D*—H···*A*
C4—H4···N4^i^	0.93	2.56	3.298 (3)	137
C2—H2···S2^ii^	0.93	2.99	3.544 (3)	119
C3—H3···S2^iii^	0.93	2.92	3.741 (3)	148
C8—H8···S1^iv^	0.93	2.97	3.738 (3)	140
C9—H9···S1^v^	0.93	2.98	3.897 (3)	169

Symmetry codes: (i) −*x*+2, −*y*+1, −*z*+1; (ii) −*x*+2, −*y*, −*z*+1; (iii) *x*−1/2, −*y*+1/2, *z*+1/2; (iv) *x*+1/2, −*y*+1/2, *z*+1/2; (v) *x*, *y*+1, *z*.

## References

[bb1] Allen, F. H. (2002). *Acta Cryst.* B**58**, 380–388. 10.1107/s0108768102003890 12037359

[bb2] Bruker (1997). *SMART*, *SAINT* and *SADABS* Bruker AXS Inc., Madison, Wisconsin, USA.

[bb3] Devel, L., Hamon, L., Becker, H., Thellend, A. & Vidal-Cros, A. (2003). *Carbohydr. Res.* **338**, 1591–1601. 10.1016/s0008-6215(03)00239-8 12860430

[bb4] Etter, M. C., Macdonald, J. C. & Wanke, R. A. (1992). *J. Phys. Org. Chem.* **5**, 191–200.

[bb5] Sheldrick, G. M. (2008). *Acta Cryst.* A**64**, 112–122. 10.1107/S0108767307043930 18156677

[bb6] Zheng, Y., Du, M., Li, J.-R., Zhang, R.-H. & Bu, X.-H. (2003). *Dalton Trans.* pp. 1509–1514

